# Physical activity is associated with reduced risk of esophageal cancer, particularly esophageal adenocarcinoma: a systematic review and meta-analysis

**DOI:** 10.1186/1471-230X-14-101

**Published:** 2014-05-30

**Authors:** Siddharth Singh, Swapna Devanna, Jithinraj Edakkanambeth Varayil, Mohammad Hassan Murad, Prasad G Iyer

**Affiliations:** 1 Division of Gastroenterology and Hepatology, Department of Internal Medicine, Mayo Clinic, 200 First Street SW, Rochester 55905MN, USA; 2 Department of Preventive Medicine, Mayo Clinic, Rochester, USA; 3 Knowledge and Evaluation Research Unit, Mayo Clinic, Rochester, MN, USA

**Keywords:** Esophageal cancer, Physical activity, Exercise, Prevention, Barrett’s esophagus

## Abstract

**Background:**

Physical activity has been inversely associated with risk of several cancers. We performed a systematic review and meta-analysis to evaluate the association between physical activity and risk of esophageal cancer (esophageal adenocarcinoma [EAC] and/or esophageal squamous cell carcinoma [ESCC]).

**Methods:**

We conducted a comprehensive search of bibliographic databases and conference proceedings from inception through February 2013 for observational studies that examined associations between recreational and/or occupational physical activity and esophageal cancer risk. Summary adjusted odds ratio (OR) estimates with 95% confidence intervals (CI) were estimated using the random-effects model.

**Results:**

The analysis included 9 studies (4 cohort, 5 case–control) reporting 1,871 cases of esophageal cancer among 1,381,844 patients. Meta-analysis demonstrated that the risk of esophageal cancer was 29% lower among the most physically active compared to the least physically active subjects (OR, 0.71; 95% CI, 0.57-0.89), with moderate heterogeneity (I^2^ = 47%). On histology-specific analysis, physical activity was associated with a 32% decreased risk of EAC (4 studies, 503 cases of EAC; OR, 0.68; 95% CI, 0.55-0.85) with minimal heterogeneity (I^2^ = 0%). There were only 3 studies reporting the association between physical activity and risk of ESCC with conflicting results, and the meta-analysis demonstrated a null association (OR, 1.10; 95% CI, 0.21-5.64). The results were consistent across study design, geographic location and study quality, with a non-significant trend towards a dose–response relationship.

**Conclusions:**

Meta-analysis of published observational studies indicates that physical activity may be associated with reduced risk of esophageal adenocarcinoma. Lifestyle interventions focusing on increasing physical activity may decrease the global burden of EAC.

## Background

Esophageal cancer is the 6^th^ most common cancer worldwide, and carries a high mortality after diagnosis following the onset of symptoms [1]. While the incidence of esophageal squamous cell cancer (ESCC) is declining in the United States, the incidence of esophageal adenocarcinoma (EAC) has increased more than 6-fold in the last three decades [2]; this has been partly attributed to the obesity epidemic. Obesity, in particular central adiposity, has been implicated in a spectrum of reflux-related esophageal diseases including erosive esophagitis, Barrett’s esophagus (BE) and EAC [3]. Routine endoscopic surveillance of patients with BE and endoscopic eradication therapy for a subset of patients with high-grade dysplasia are recommended [4]. However, this strategy is expensive and limited by suboptimal adherence and access. Chemopreventive strategies using aspirin, statins or proton-pump inhibitors require a large number of patients be treated to prevent a single cancer, making it difficult to ascertain risk-benefit ratio and cost-effectiveness [3,5,6].

For non-tobacco users, diet and physical activity are the most important modifiable determinants of cancer risk [7]. Physical activity has been associated with a reduced incidence and mortality from certain cancers, including proximal and distal colorectal cancer [8], gastric cancer [9], breast and endometrial cancers [7,10]. The protective effect of physical activity against cancer is possibly mediated by counteracting the adverse carcinogenic effects of obesity, improving insulin sensitivity and decreasing systemic inflammation leading to favorable immunomodulation [11,12]. There have been several studies reporting an inverse association between physical activity and risk of esophageal cancer [13,14], but results have been inconsistent [15,16]. Several systematic reviews on physical activity and cancer prevention have not addressed esophageal cancer risk [7,17].

To better understand the relationship between physical activity and esophageal cancer risk, in particular, the risk of EAC, we performed a systematic review with meta-analysis of all studies that investigated the association between physical activity and risk of esophageal cancer in adults.

## Methods

This systematic review is reported according to the Preferred Reporting Items for Systematic reviews and Meta-Analyses (PRISMA) guidelines [18]. The process followed *a priori* established protocol (available upon request).

### Search strategy and selection criteria

**Figure 1:**
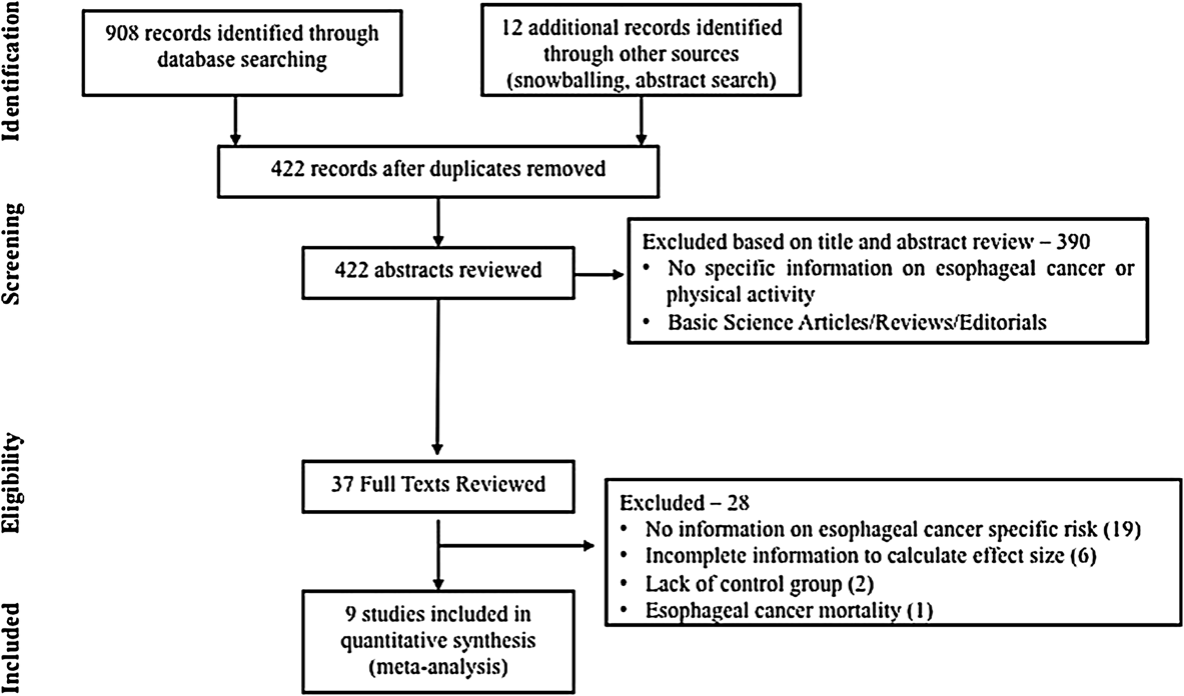
Flow diagram summarizing study identification and selection.

A systematic literature search of PubMed (1966 through February 1, 2013), Embase (1988 through February 1, 2013) and Web of Science (1993 through February 1, 2013) databases was conducted to identify all relevant studies on the relationship between physical activity and risk of esophageal cancer. Studies considered in this meta-analysis were observational studies or randomized controlled trials (RCTs) that met the following inclusion criteria: (1) evaluated and clearly defined physical activity (recreational or occupational), (2) reported risk of esophageal cancer (EAC and/or ESCC) and (3) reported relative risk (RR) or odds ratio (OR) with 95% confidence intervals (CI) of the association between physical activity and esophageal cancer risk, or provided data for their calculation. A combination of key words was used in the search: (exercise OR physical activity OR walking OR motor activity) AND (esophagus) AND (cancer OR neoplasm OR carcinoma). Expansion of the search to combination of physical activity and cancer did not result in identification of any additional articles. Then, per the protocol-defined study inclusion and exclusion criteria, two authors (S.S. and J.E.V.), independently reviewed the title and abstract of studies identified in the search to exclude studies that did not investigate the association between physical activity and the risk of esophageal cancer. The full text of the remaining articles was examined to determine whether it contained relevant information. Next, the bibliographies of the selected articles, as well as review articles on the topics were manually searched for additional articles. We also searched conference proceedings of major gastroenterology (Digestive Diseases Week, United European Gastroenterology Week, American College of Gastroenterology annual meeting) and oncology conferences (American Society of Clinical Oncology annual meeting and Gastrointestinal Research Forum; European Society of Medical Oncology annual meeting and World Congress on Gastrointestinal Cancer) from 2005–2012 for studies that had been published only in the abstract form. Inclusion was not otherwise restricted by study size, language or publication type. Studies that examined only the association between physical activity and cancer-related mortality were excluded. When there were multiple publications from the same population, only data from the most comprehensive report were included. The flow diagram summarizing study identification and selection is shown in Figure 1.

### Data abstraction

After study identification, data on study and patient characteristics, exposure and outcome assessment, potential confounding variables and estimates of association were independently abstracted onto a standardized form by two authors (S.S. and S.D.). The following data were collected from each study: (a) study and patient characteristics: primary author, time period of study/year of publication, country of the population studied, age, sex, body mass index; (b) physical activity measurement: physical activity domain assessed (recreational and/or occupational) and instrument used for measurement (whether valid and reliable); (c) esophageal cancer ascertainment: histology-specific relationship (EAC and ESCC), method of outcome ascertainment (self-report, cancer registry with or without independent validation); (d) potential confounding variables accounted for: age, sex, obesity, race/ethnicity, cigarette smoking, alcohol intake, family history of esophageal cancer, other medication use (aspirin/non-steroidal anti-inflammatory drugs [NSAIDs], statins, proton-pump inhibitors) and (e) estimates of association between physical activity and esophageal cancer risk: adjusted RR or OR and 95% confidence interval (CI).

If a study combined the two physical activity domains (recreational and occupational) into a single measure, then the effect estimate for the combined measure result was used for the primary meta-analysis. If a study reported the effect estimates for two or more domains of physical activity separately, then we pooled the results into a single measure using fixed effects model of meta-analysis. If a study reported the effect of physical activity at multiple periods or ages and over the lifetime, we used the lifetime result. For all studies, we used the result that compared the most active group with the least active group (reference group). For studies in which the most active group was used as the reference group, we inverted the effect size and 95% CI. To estimate the dose–response relationship, using the least active group as reference, we measured the association between the middle tertile/quartile and reference as well as the association between the highest tertile/quartile and reference, and analyzed whether the difference between these estimates was significantly different. Conflicts in data abstraction were resolved by consensus, referring back to the original article.

### Quality assessment

The risk of bias in included studies was assessed by two authors independently (S.S. and J.E.V.), using the methodology suggested by Boyle et al. [8]. Briefly, we used a three-item checklist to identify whether studies were at low or high risk of bias, based on: (a) study design – low risk of bias if studies were cohort or population-based case–control studies, and high risk of bias if hospital-based case–control or exclusively cancer registry-based; (b) instrument used to measure physical activity – low risk of bias if instrument was reliable as shown in index study or related study, and high risk of bias if not reported; (c) key variables adjusted or accounted for: age, sex and obesity. If a study adjusted, matched or accounted for the potential confounding effect of age, sex and obesity in their analysis, then those studies were considered to be at low risk of bias, otherwise they were considered to be at high risk of bias. Overall, if a study was deemed to be at low-risk of bias across all these domains, then it was considered a high-quality study; if the study was at high-risk of bias across one or more of the three domains, then it was considered low-quality study [8]. The overall agreement between the two reviewers for the final determination of each study was excellent (Cohen’s κ = 0.86), and disagreements were resolved by consensus.

### Outcomes assessed

#### Primary outcome

The primary analysis focused on assessing the association between physical activity and the risk of (a) overall esophageal cancer, as well as by (b) histological subtypes – EAC and ESCC.

#### Subgroup analysis

*A priori* hypotheses to examine robustness of association and explain potential heterogeneity in the direction and magnitude of effect among different observational studies included location of study (Western population v. Asian population), study design (case–control v. cohort) and study quality (high v. low). In addition, we measured the impact of recreational and occupational activity domains separately, since the former is the modifiable aspect of energy expenditure.

### Statistical analysis

We used the random-effects model described by DerSimonian and Laird to calculate pooled OR and 95% CI [19]. Since outcomes were relatively rare, OR were considered approximations of RR. Adjusted OR reported in studies was used for analysis to account for confounding variables. We assessed heterogeneity between study-specific estimates using the inconsistency index [20]. To estimate what proportion of total variation across studies was due to heterogeneity rather than chance, I^2^ statistic was calculated. In this, a value of <30%, 30%-60%, 61%-75% and >75% were suggestive of low, moderate, substantial and considerable heterogeneity, respectively [21]. Once heterogeneity was noted, between-study sources of heterogeneity were investigated using subgroup analyses by stratifying original estimates according to study characteristics (as described above). In this analysis also, a p-value for differences between subgroups of <0.10 was considered statistically significant (i.e., a value of p < 0.10 suggested that stratifying based on that particular study characteristic partly explained the heterogeneity observed in the analysis). Given the small number of studies identified in our analysis, statistical tests for assessing publications bias were not performed [22]. All p-values were two tailed. For all tests (except for heterogeneity), p < 0.05 was considered statistically significant. All calculations and graphs were performed using Comprehensive Meta-Analysis (CMA) version 2 (Biostat, Englewood, NJ).

## Results

### Study flow

From 422 unique studies identified using the search strategy, 9 studies met the inclusion criteria [13-16,23-27]. These studies reported on the association between physical activity and 1,871 cases of esophageal cancer among 1,381,844 patients. During the peer review process, with an updated search till May 1, 2014, an additional hospital case–control study from India was identified with 704 cases of ESCC [28]. No relevant RCTs were identified. The coefficient of agreement between the two reviewers for study selection was excellent (Cohen’s κ = 0.82). Six studies on dietary or socioeconomic risk factors for cancer mentioned assessing physical activity as a covariate but did not specifically measure or report association between physical activity and esophageal cancer per se [29-34]; four of these studies were published more than 15 years ago and hence, data was not accessible; additional data could not be obtained from contacting authors of two recent studies and hence, these were excluded. Two studies did not have an appropriate control group [35,36]. In a Dutch cohort, de Jonge and colleagues compared the mean physical activity levels in patients with EAC, ESCC and gastric cardia adenocarcinoma, but there was no referent population to allow calculation of a risk estimate [35]. The same group compared differences in physical activity levels in patients with BE with and without EAC and did not observe any significant differences, but an estimate of EAC risk among the most physically active to the least physically active was not possible [36]. One study reported the association between physical activity and mortality from esophageal cancer, and was excluded [37].

### Characteristics and quality of included studies

#### Baseline characteristics

The characteristics of these studies are shown in Table 1. The earliest cohort study recruited patients starting in 1978 and latest completed recruitment in 2007, with mean reported follow-up ranging from 6 to 18.8 years. Seven studies were performed in the Western population (5 in North America, 2 in Europe) [13-16,23,25,26] and two studies were performed in Asian population [24,27]. Four studies were performed exclusively in men [13,23-25]. In three studies, recreational (with or without household) physical activity was the only measured domain [16,23,24]; in three studies, only occupational physical activity was inferred based on the job-title [14,26,27]. Physical activity was assessed using self-administered questionnaire in most of the studies, and was based on a combination of intensity, duration and frequency of recreational physical activity. Of the nine studies, three reported exclusively on risk of EAC [14,15,26], and one reported exclusively on the risk of ESCC [27]; four studies reported on risk of esophageal cancer with no separate information on risk by histological-subtype [13,23-25].

**Table 1:** Baseline characteristics of the included studies

**First Author, Year of Publication**	**Study Setting; Location**	**Time Period; Follow-up**	**Total no. of participants**	**No. of esophageal cancer cases (EAC/ESCC)**	**Physical activity domain**	**Physical activity measurement; valid/reliable**	**Outcome measurement**	**Variables adjusted for**
***Cohort Studies***								
Huerta, 2010 [15]	Popula`tion-based; Europe (European Prospective Investigation into Cancer and Nutrition); 25-70y old men and women	Recruitment: 1992–2000; F/U: 8.8y	420,449	Total: 85	Recreational + Occupational (separate also)	Self-administered questionnaire; Yes	Central Cancer Registries; health insurance records, cancer and pathology hospital registries, active follow-up	1,2,3,5,6,7,8
				EAC: 85				
				ESCC: NA				
Leitzmann, 2009 [16]	Population-based; USA (NIH-AARP Diet and Health Study); 50-71y old men and women	Recruitment: 1995–1996; F/U: 8y	487,732	Total: 523	Recreational	Self-administered questionnaire; Yes	Central Cancer Registry	1,2,3,4,5,6,7,8,9
				EAC: 149				
				ESCC: 374				
Wannamethee, 2001 [23]	Population-based; England (British Regional Heart Study); 40–59 y old men	Recruitment: 1978–1980; F/U 18.8y	7,588	Total: 65	Recreational	Self-administered questionnaire; Yes	Central Cancer Registry, death certificates, postal follow-up	1,2,3,5,6,9
				EAC: NR				
				ESCC: NR				
Yun, 2008 [24]	Population-based; Korea (National Health Examination Program); >40y old men	Recruitment: 1996; F/U 6y	444,963	Total: 293	Recreational	Self-administered questionnaire; Yes	Central Cancer registry	1,2,3,4,5,6,7,10
				EAC: NR				
				ESCC: NR				
***Case–control Studies***								
Balbuena, 2008 [26]	Hospital-based; Canada	2002-2004	327	Total: 57	NR	NR	NR	NR
				EAC: 57				
				ESCC: NA				
Brownson, 1991 [25]	Cancer Registry; USA; >20y men	1984-1989	17,147 (all cancerpatients)	Total: 237	Occupational	Job-title based; No	Central Cancer Registry	1,2,3,5
				EAC: NR				
				ESCC: NR				
Etemadi, 2012 [27]	Hospital-based; Iran	2003-2007	871	Total: 300	Occupational	Self-administered questionnaire; No	Gastroenterology Clinic, based on histological validation	1,2,5,8,9
				EAC: NA				
				ESCC: 300				
Parent, 2010 [13]	Population-based; Canada; 35-70y old men	1979-1985	784	Total: 99	Recreational + Occupational (separate also)	Interviewer-administered questionnaire; No	Central Cancer Registry, with independent validation	1,2,3,4,5,6,7,9
				EAC: NR				
				ESCC: NR				
Vigen, 2006 [14]	Population-based; USA; 30-74y old men and women	1992-1997	1,983	Total: 212	Occupational	Job-title based; No	Central Cancer Surveillance Program	1,2,3,4,5,9
				EAC: 212				
				ESCC: NA				
Dar 2013* [28]	Hospital-based; India	2008-2012	2,367	Total: 703	Occupational	Job-title based; No	Hospital oncology clinic, based on histological validation	1,2,4,5,6,7,9
				EAC: NA				
				ESCC: 703				

*additional study identified during the peer review process with an updated search (1-Age, 2-Sex, 3-Obesity (BMI, Weight), 4-Race/Ethnicity, 5- Smoking, 6-Alcohol, 7-Dietary factors, 8-Family history of esophageal cancer, 9-Education and Socioeconomic status, 10-Diabetes) [Abbreviations: *EAC*-Esophageal adenocarcinoma; *ESCC*-Esophageal squamous cell carcinoma; *F/U*-Follow-up; *NA*-Not applicable; *NR*-Not reported].

#### Quality assessment

Four observational studies (all cohort studies) were at low-risk of bias based on study design, exposure ascertainment and adjusting for key confounding variables, and were deemed to be of high quality (Table 2) [15,16,23,24]. The included studies variably accounted for other potential confounders: smoking (8/9), obesity (7/9), alcohol use (5/9) and family history of esophageal cancer (3/9); none of the studies adjusted for gastroesophageal reflux symptoms. Socioeconomic status, which appears to have inverse association with physical activity was accounted for in 5/9 studies. For outcome ascertainment, most studies relied on record linkage through the cancer registry (with or without review of death certificates and pathology databases), or review of medical records. In all these studies, a temporal relation between exposure and outcomes was established – physical activity preceded esophageal cancer by at least 1 year, and usually longer periods.

**Table 2:** Quality assessment of included studies

	**Bias in study design**	**Bias in instrument to measure physical activity**	**Bias in accounting for confounding variables**	**Overall quality of study**
***Cohort studies***				
Huerta [15]	Low	Low	Low	High
Leitzmann [16]	Low	Low	Low	High
Wannamethee [23]	Low	Low	Low	High
Yun [24]	Low	Low	Low	High
***Case–control Studies***				
Balbuena [26]	High	High	High	Low
Brownson [25]	High	High	Low	Low
Etemadi [27]	High	High	High	Low
Parent [13]	Low	High	Low	Low
Vigen [14]	Low	High	Low	Low

Briefly, we used a three-item checklist to identify whether studies were at low or high risk of bias, based on: (a) study design – low risk of bias if cohort or population-based case–control studies, and high risk of bias if hospital-based case–control or exclusively cancer registry-based; (b) instrument used to measure physical activity – low risk of bias if instrument valid and reliable as shown in index study or related study, and high risk of bias if not reported; (c) key variables adjusted or accounted for: if a study adjusted, matched or accounted for the potential confounding effect of age, sex and obesity in their analysis, then those studies were considered to be at low risk of bias, otherwise they were considered to be at high risk of bias. Overall, if a study was deemed to be at low-risk of bias across all these domains, then it was considered a high-quality study, otherwise it was considered a low-quality study.

### Physical activity and risk of esophageal cancer

#### Overall risk of esophageal cancer

**Figure 2:**
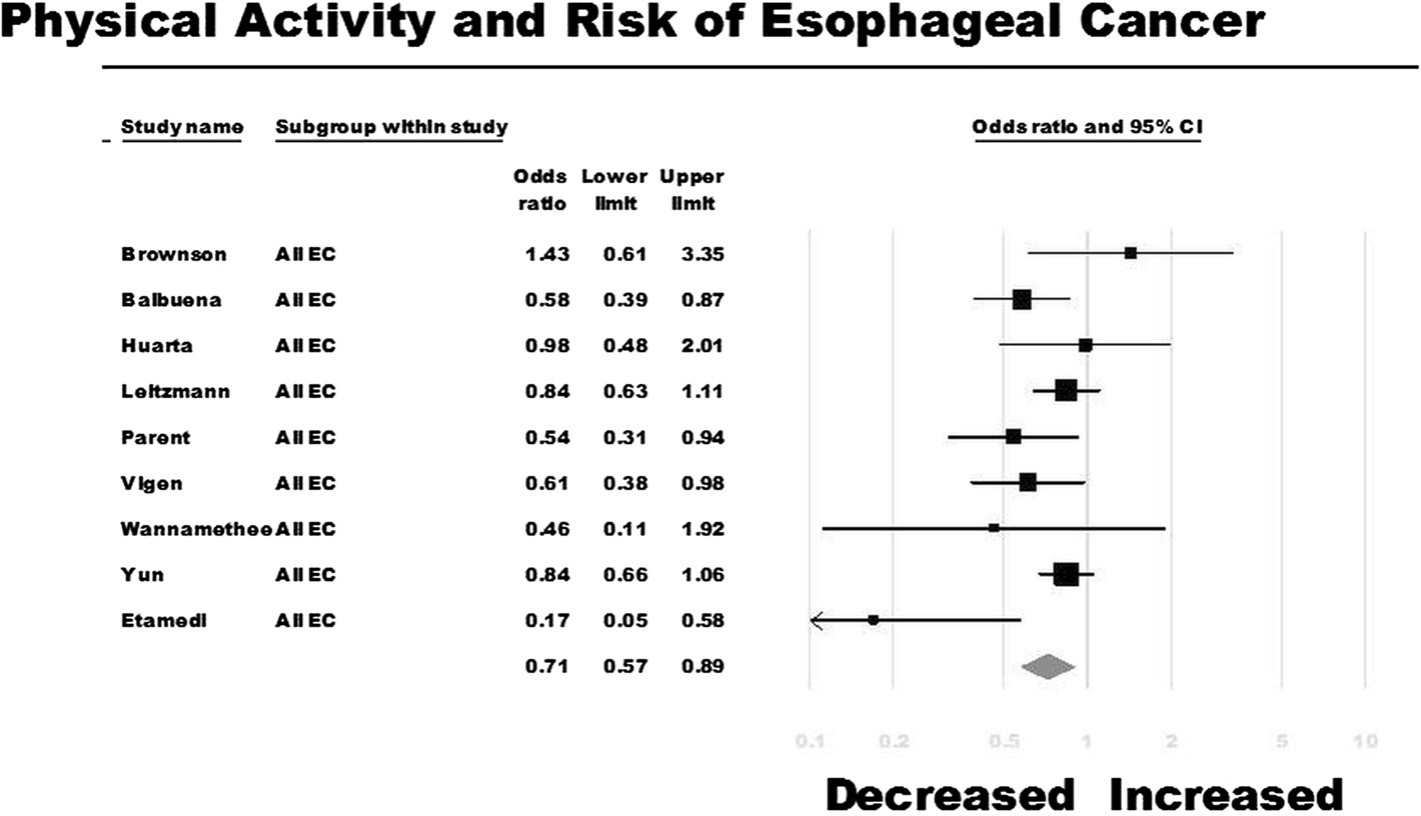
Physical activity and risk of esophageal cancer.

Of the nine studies identified, four reported a statistically significant inverse association between overall physical activity and esophageal cancer risk [13,14,26,27]. On meta-analysis, risk of esophageal cancer was 29% lower among the most physically active people as compared with the least physically active people (OR, 0.71; 95% CI, 0.57-0.89) (Figure 2). There was moderate heterogeneity observed across studies (I^2^ = 47%).

#### Risk of esophageal adenocarcinoma

**Figure 3:**
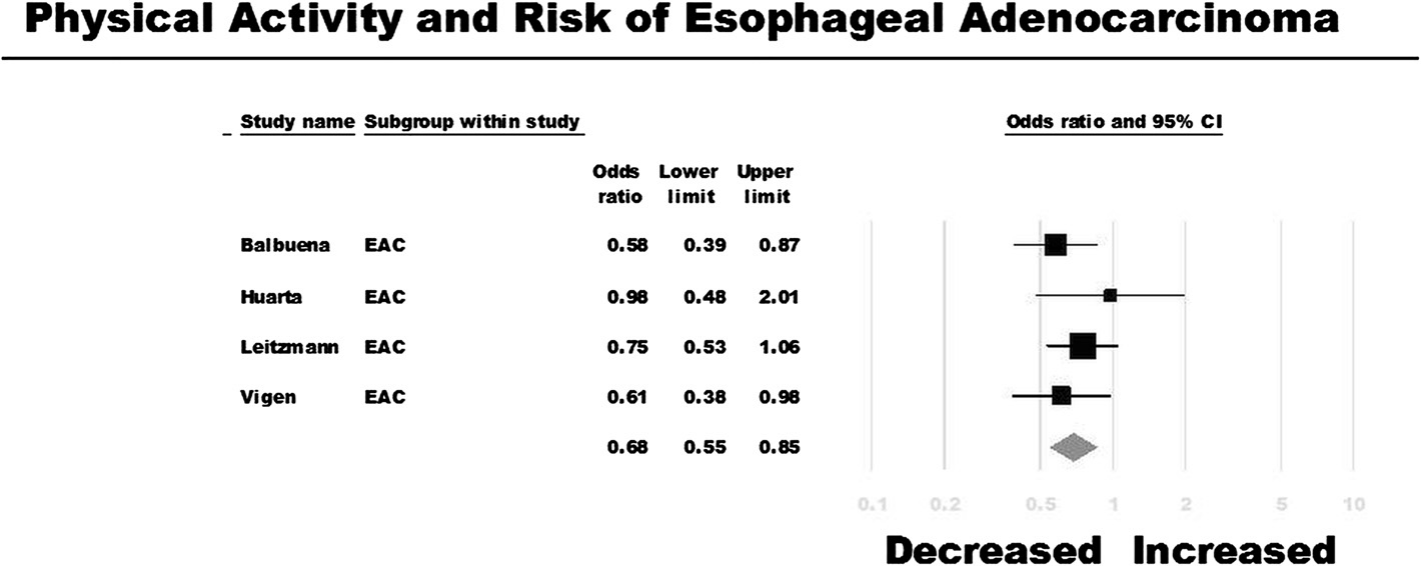
Physical activity and risk of esophageal adenocarcinoma.

Of the four studies identified [14-16,26], two reported a statistically significant inverse association between physical activity and EAC risk [14,26]. On meta-analysis, risk of EAC was 32% lower among the most physically active people as compared with the least physically active people (OR, 0.68; 95% CI, 0.55-0.85) (Figure 3). There was minimal heterogeneity observed across studies (I^2^ = 0%).

#### Risk of esophageal squamous cell carcinoma

Only two studies reported the association between physical activity and risk of ESCC [16,27]. One of them, performed in Iran, observed a strong inverse association [27], whereas the other, performed in the United States, reported a null association [16]. During the peer review process, another low quality, case–control study published after data of search was identified. This study performed in India reported a 5-fold higher risk of ESCC in patients with the highest level of occupational physical activity. On meta-analysis, there was no association between physical activity and risk of ESCC (OR, 1.10; 95% CI, 0.21-5.64), albeit with considerable heterogeneity (I^2^ = 95%).

### Subgroup and sensitivity analyses

#### Subgroup analysis

On sub-group analysis, the association between physical activity and risk of esophageal cancer was stable across case–control and cohort studies, and across Western and Asian population (Table 3). On analysis by domain of physical activity, recreational physical activity, the potentially modifiable component of physical activity, was associated with a decreased risk of esophageal cancer (OR, 0.79; 95% CI, 0.67-0.93; I^2^ = 0%).

**Table 3:** Sub-group analyses, as well as dose–response relationship, on the association of physical activity and esophageal cancer risk

**Groups**	**Categories**	**No. of Studies**	**Adjusted OR**	**95% CI**	**Heterogeneity within groups (I**^**2**^**)**	**P-difference between groups**
Study Design	Case–control	5	0.59	0.40-0.88	51	0.11
	Cohort	4	0.84	0.71-1.00	0	
Study Location	Asian	2	0.43	0.09-2.00	84	0.51
	Western	7	0.72	0.58-0.89	18	
Study Quality	High	4	0.84	0.71-1.00	0	0.11
	Low	5	0.59	0.40-0.88	51	
Dose–response	Middle tertile^a^	5	0.88	0.70-1.10	19	0.41
	Highest tertile^a^	5	0.76	0.60-0.97	0	

^a^using least active people as reference category [Abbreviations: *EAC*-Esophageal adenocarcinoma; *ESCC*-Esophageal squamous cell carcinoma].

#### Dose–response relationship

A non-significant trend towards an inverse dose response relationship between physical activity and esophageal cancer risk was observed. Using the least active group as reference, people in the middle tertile or 2^nd^ quartile of physical activity had a non-statistically significant 12% lower risk of esophageal cancer (5 studies; OR, 0.88; 95% CI, 0.70-1.10; I^2^ = 19%) [14-16,23,25]. In comparison, the most physically active people (highest tertile of physical activity or 4^th^ quartile) had a 24% lower risk of esophageal cancer (5 studies; OR, 0.76; 95% CI, 0.60-0.97; I^2^ = 0%).

#### High-quality studies

On restricting analysis to the four high-quality studies [15,16,23,24], we observed that physical activity is associated with a 16% lower risk of esophageal cancer, though this association did not reach pre-specified statistical significance (OR, 0.84; 95% CI, 0.71-1.00; p = 0.05). The results were consistent across studies (I^2^ = 0%).

#### Sensitivity analysis

To assess whether any one study had a dominant effect on the summary OR, each study was excluded and its effect on the main summary estimate was evaluated. While no study significantly affected the summary estimate, exclusion of the study by Etemadi and colleagues on the association between physical activity and risk of ESCC resulted in resolution of the previously observed marked heterogeneity in the analysis. The favorable and strong effect sizes observed in this single study were causing heterogeneity in the strength, but not the direction, of overall association. On analysis after excluding this study, the summary estimate remained significant (OR, 0.76; 95% CI, 0.64-0.89) and minimal heterogeneity was observed in the analysis (I^2^ = 15%).

Given the small number of studies identified in our analysis, statistical tests for assessing publications bias were not performed.

## Discussion

Based on the evidence derived from this systematic review and meta-analysis of published studies, increasing physical activity is associated with a 29% lower risk of esophageal cancer, after adjustment for important confounders including age, obesity and other risk factors for esophageal cancer. After exclusion of one study, which was responsible for heterogeneity, a 24% reduction in risk of esophageal cancer with increasing physical activity was a more conservative and consistent estimate. Specifically, the risk reduction was primarily seen in risk of EAC (32% lower risk amongst the most physically active people than the least active people), which has been strongly associated with obesity-associated chronic inflammation. We did not observe a significant association between physical activity and risk of ESCC, though the number of studies was small (3 studies). The analysis was considerably limited due to the conflicting observations from two of the included studies, with one showing a strong inverse association (OR, 0.16) and another showing a strong direct association (higher risk of ESCC with increasing occupational physical activity) (OR, 5.65). The results were stable across cohort and case–control studies in both Asian and Western population. Importantly, recreational physical activity, the potentially modifiable component of energy expenditure, was independently associated with reduced risk of esophageal cancer, with a trend towards a dose–response relationship.

With the high incidence and poor prognosis associated with esophageal cancer, cost-effective strategies aimed at preventing esophageal cancer are highly desirable. While chemopreventive strategies are attractive, currently, their cost-effectiveness and risk-benefit ratio is difficult to ascertain. This EAC risk modification observed with physical activity is comparable to the 30-40% risk reduction seen with aspirin/NSAID and statin use [3,5]. Moreover, this point estimate for EAC risk reduction with physical activity is comparable to the more established 21%, 24% and 27% reduction in risk for gastric [9], colorectal [38] and endometrial cancer [10], respectively. Previous systematic reviews have summarized evidence from epidemiological studies on the association between physical activity and gastrointestinal cancer prevention and mortality [26,39]. However, in those reviews, only a single electronic database was searched resulting in some missed studies; there was no quality appraisal of current literature on this topic. A quantitative synthesis of the literature to calculate a summary estimate was not performed for the overall association or for sub-groups. In its 2007 report on the role of food, nutrition and physical activity, the World Cancer Research Fund and American Institute of Cancer Research did not make any statement on the role of physical activity in decreasing esophageal cancer risk [7].

Physical activity can modify the risk of cancer through several proposed mechanisms. Metabolic syndrome and insulin resistance have been associated with increased risk of cancer, particular EAC [40-43]. This is mediated by adipokines and cytokines released by metabolically active visceral fat, which result in chronic hyperinsulinemia and increase risk of insulin-like growth factor-mediated carcinogenesis [44]. Exercise decreases visceral fat, lowering the level of carcinogenic adipocytokines, improves insulin sensitivity and reduces fasting insulin and C-peptide levels, and may decrease insulin-like growth factor-1 [12]. Physical activity has been shown to decrease chronic inflammation in intervention trials decreasing interleukin-6 and tumor necrosis factor-α, independent of weight loss [12]. Additionally, exercise has been shown to have immunomodulatory effects, improving innate and acquired immune response, promoting tumor surveillance [12,45]. Studies have also shown that aerobic exercise can decrease oxidative stress and enhance DNA repair mechanisms, decreasing carcinogenesis [45]. Physically active individuals also have higher sunlight exposure and consequently, increased vitamin D levels, which may modify cell proliferation cascades [46].

### Strengths and limitations

The strengths of this analysis include (a) comprehensive assessment of the association between physical activity and overall and histological-subtype specific risk of esophageal cancer; (b) analyses accounting for the effect of potential confounders particularly age, obesity and other risk factors for esophageal cancer such as smoking and alcohol use, in summarizing risk estimates by using the maximally adjusted point estimates from each study; (c) incorporating the effect of both recreational and occupational physical activity, independently on esophageal cancer risk; (d) assessment of a dose–response relationship; (e) sensitivity analyses based on study quality and (f) inclusion of all available studies and not restricting analysis based on study design, publication type or language, and hence, being at low risk for selection or publication bias.

There are several limitations in our study. First, the meta-analysis included only observational studies. No randomized controlled trials have been performed to explore this association. Despite adjusting for numerous covariates, it is not possible to eliminate the potential of residual confounding. It is possible that the observed decreased risk of esophageal cancer seen in more physically active people may relate to a ‘healthy user’ bias [47]. Physically active people may be more compliant with preventive health measures, as compared to patients who are not physically active. Physically inactive, and potentially poorly compliant, patients may have other unhealthy lifestyle practices predisposing them to esophageal cancer. While most of the studies accounted for some such lifestyle factors such as obesity, smoking and alcohol use, socioeconomic status was not consistently accounted for. Socioeconomic status interacts with both exposure (level of physical activity) and outcome (risk of esophageal cancer), and may have contributed to unmeasured confounding. Additionally, none of the studies adjusted for the presence of reflux symptoms or erosive esophagitis. Moderate, but not intense, physical activity has been associated with decrease in reflux symptoms in obese subjects, but not in non-obese subjects [48,49]. Hence, what is perceived as a physical activity-mediated effect may indeed represent a sum of events and interactions, which modify esophageal cancer risk in these physically active people. That said, an independent protective association was also observed on restricting analysis to high quality studies. Second, we could not assess for publication bias due to the small number of studies on this topic. Six studies on dietary or socioeconomic risk factors for cancer measured physical activity as a covariate, but did not measure the association between physical activity and esophageal cancer; one study measured the association but did not provide sufficient data on effect size, suggesting the presence of reporting and probable publication bias [31]. Third, moderate heterogeneity was observed in the overall analysis, which was primarily attributable to a single study, which showed a strong inverse association between physical activity and risk of ESCC [27]. Fourth, no credible inference can be drawn on the association between physical activity and risk of ESCC, due to the small number of low quality studies with markedly conflicting results. The timing, intensity and domain of physical activity may influence its association with health outcomes, but a detailed assessment of all these factors was not reported in individual studies. Another potential limitation that particularly applies to case–control studies evaluating cancer risk is recall bias, especially since most of these studies used a self-administered questionnaire to measure physical activity. However, on sub-group analysis, pooled analysis of prospective cohort studies reported a similar association between physical activity and esophageal cancer risk, and there was no significant difference in risk estimates between case–control and cohort studies.

## Conclusions

Based on this systematic review and meta-analysis of all observational studies, we observed that the risk of esophageal cancer, in particular EAC, may be lower among the most physically active people as compared with the least physically active people. Hence, EAC risk reduction may be an additional benefit to a myriad of health benefits with being physically active, which include cardiovascular, metabolic and psychological wellbeing. Currently, it is unclear what is the ideal type, intensity, frequency and time period of physical activity that may modify cancer risk. An ongoing, 24-week randomized controlled trial of moderate-intensity aerobic and resistance training in overweight males with BE to estimate its effect on risk of progression to EAC may help shed more light on this topic [50]. For now, in the absence of interventional studies of physical activity on cancer risk, the American Cancer Society recommends “adopting a physically active lifestyle” and suggests that “adults engage in at least 150 minutes of moderate intensity or 75 minutes of vigorous intensity activity each week, or an equivalent combination, preferably spread throughout the week” [51].

## Abbreviations

BE: Barrett’s esophagus; CI: Confidence intervals; EAC: Esophageal adenocarcinoma; ESCC: Esophageal squamous cell cancer; OR: Odds ratio.

## Competing interests

The authors declare that they have no competing interests.

## Authors’ contributions

SS and PGI were involved in study concept and design; SS, SD and JEV were involved in acquisition of data; SS, MHM and PGI were involved in statistical analysis and interpretation of data; SS and SD were involved in drafting of the manuscript; JEV, MHM and PGI were involved critical revision of the manuscript for important intellectual content. All authors read and approved the final manuscript.

## Pre-publication history

The pre-publication history for this paper can be accessed here:

http://www.biomedcentral.com/1471-230X/14/101/prepub
